# LMP-PM: a lightweight multi-path pruning method for plant leaf disease recognition

**DOI:** 10.3389/fpls.2026.1737464

**Published:** 2026-02-26

**Authors:** Jing Hua, Fendong Zou, Yuanhao Zhu, Jize Deng, Ruimin He

**Affiliations:** School of Software, Jiangxi Agricultural University, Nanchang, China

**Keywords:** convolutional neural network, deep learning, lightweight, LMP-PM, plant disease identification

## Abstract

Plant leaf diseases pose a significant threat to plant growth and productivity, necessitating accurate and timely identification. While high-performance deep learning models exist, their complexity often hinders deployment in real-world, resource-constrained agricultural settings. To address the need for efficient and accurate plant disease identification, we developed a novel lightweight approach named the Lightweight Multi-Path Pruning Method (LMP-PM). LMP-PM offers flexible lightweight optimization, configurable via pruning parameters and path expansion ratios, enabling users to balance significant reductions in model parameters and FLOPs against potential inference time increases, thereby tailoring model size, performance, and real-time needs to specific application scenarios. Specifically, we first constructed an original, high-performance, and complex model (OMNet) incorporating various structures and a three-branch parallel module (TBP block). We then applied LMP-PM to OMNet to perform lightweight processing, resulting in several lightweight models. Through extensive experimentation, we identified the optimal model that balances performance and complexity, which we named LMNet (Lightweight Multi-Path Network). LMNet demonstrates remarkable efficiency, utilizing only 5.69% of the parameters and 3.80% of the FLOPs of OMNet. Despite this substantial reduction in complexity, LMNet achieved superior accuracy: 99.23% on the Plant Village dataset, representing an improvement of 0.58% over OMNet, and 87.27% on the AI 2018 Challenger dataset, surpassing OMNet by 1.91%. These results highlight that LMP-PM successfully creates highly efficient models like LMNet, which not only drastically reduce computational resources but also improve classification accuracy. This flexibility and enhanced performance make LMNet particularly suitable for real-time plant disease identification in resource-constrained environments, offering a practical and effective solution for agricultural applications.

## 1 Introduction

Plant leaf diseases are multifactorial and pose a serious threat to plant productivity (Xiao et al., 2024). These diseases can lead to reduced yields, plant death, and significant losses, thereby hindering overall food production. Accurate identification of leaf diseases is crucial for mitigating their impact on plant production (Khanna et al., 2024; Swaminathan and Vairavasundaram, 2024; Tang L. et al., 2024). However, the identification of leaf diseases presents challenges due to the wide variety of disease types, the difficulty in recognition, and the short disease cycles. Traditionally, the identification of plant diseases has relied on field sampling conducted by specialists, who then visually observe or analyze samples in a laboratory to determine the specific types of diseases present. However, this method often faces limitations due to a lack of available experts, leading to inefficient time management and the potential to miss critical treatment windows (Pal and Kumar, 2023; Sahu and Pandey, 2023; Shi et al., 2023; Deng et al., 2024).

In recent years, with the advancement of artificial intelligence technology, deep learning and computer vision techniques have been widely used for the identification of plant diseases (Parthiban et al., 2023; Salamai et al., 2023; Wu et al., 2023; Dong et al., 2024; Li H. et al., 2024; Wang H. et al., 2024). Techniques such as image classification (Cheng et al., 2023; Lin et al., 2024; Tang W. et al., 2024), object detection (Denarda et al., 2024; Hernández et al., 2024; Yu X. et al., 2024), and semantic segmentation (Heidari et al., [[NoYear]]; Jain et al., [[NoYear]]; Dai et al., 2024) have gained prominence in this field. Currently, deep learning applications in the identification of plant leaf diseases primarily focus on two key directions. The first direction involves the development of large models (Zhang T. et al., 2023; Kuska et al., 2024) and multimodal fusion (Yu H. et al., 2024; Zhang et al., 2024). However, large models and multimodal tasks often rely on high-performance computing equipment, which is typically expensive. Consequently, agricultural practitioners in real-world settings frequently lack the purchasing power for such equipment, limiting the applicability of these methods. The second trending direction is the use of lightweight models (Hua et al., 2023; Huang et al., 2023; Wang B. et al., 2024; Xie et al., 2024). These models do not require expensive hardware and can be effectively applied in practical agricultural production. This advantage has facilitated their wider application and promotion within the agricultural sector. Lightweight models can run on resource-constrained devices such as smartphones, edge computing devices, and small embedded systems, providing quick responses that meet the demands of real-time agricultural production monitoring (Yongda et al., 2024).

Existing methods for lightweighting plant leaf disease models often achieve their goals by introducing new modules or functions to replace existing, more complex components. For instance, Li R. et al. (2024) introduced the ECIoU_Loss (EfficiCLoss Loss) function, replacing the original CIoU_Loss. This modification resulted in a 50.2% reduction in model size and a significant 43.1% decrease in FLOPs, achieving a best accuracy of 87.5% in corn leaf disease identification tasks. Similarly, Sandesh et al. (Bhagat et al., 2024) replaced the standard convolution in Vgg16 with MDsConv, which reduced model parameters by 60% while achieving an accuracy of 94.14%, surpassing the performance of the standard Vgg16 model. Additionally, Siyu et al. (Quan et al., 2024) utilized partial convolution and point-wise convolution techniques to replace traditional deep convolutions, thereby reducing computational complexity and attaining a best accuracy of 99.04% on the PlantVillage dataset. Xing et al. (2024) adopted the lightweight network structure ShuffleNetV2 to replace the traditional backbone network Xception in DeepLabv3+. The improved DeepLabv3+ achieved an average pixel accuracy and mean intersection over union of 95.84% and 96.87%, respectively, with a detection rate and weight file size superior to other algorithms. Lastly, Zhang P. et al. (2023) employed mixed lightweight convolution and spatial pyramid dilated convolution to replace standard convolutions. Their proposed LW-Segnet and LW-Unet models achieved higher F1 scores and intersection-over-union values in seedling detection and cross-variety row segmentation while reducing model parameters.

The lightweight methods described above have successfully enhanced model performance while reducing the complexity of the original models, demonstrating the feasibility of lightweight models in the identification of plant leaf diseases. However, these approaches primarily involve structural replacements of the original model without considering the effects of model pruning and path expansion on both model complexity and performance, often resulting in limited degrees of lightweighting. To address this research gap, we developed a new lightweighting approach named the Lightweight Multi-Path Pruning Method (LMP-PM). LMP-PM significantly reduces the complexity of the original model while allowing for selective control of the degree of lightweighting through the adjustment of pruning parameters and path expansion ratios, thereby catering to varying task requirements. Specifically, we initially constructed an original model (OMNet), which boasts a high level of complexity and incorporates various structures, including a three-branch parallel module (TBP block) designed to optimize performance. Subsequently, we applied LMP-PM to perform lightweight processing on OMNet, resulting in several lightweight models. We selected the best one and named it LMNet. The contributions of this paper are as follows:

LMP-PM: We proposed a new lightweight method, the Lightweight Multi-Path Pruning Method (LMP-PM), which selectively controls the degree of lightweight processing.

OMNet: We introduced a novel base model, OMNet, which incorporates various structures and exhibits high performance and complexity.

LMNet: LMNet is derived from OMNet through the lightweight processing of LMP-PM. It features fewer parameters while achieving superior performance in the identification of leaf diseases.

TBP Block: We developed a new three-branch parallel module designed for multi-scale extraction of plant leaf disease characteristics, effectively enhancing recognition performance.

## 2 Materials and methods

### 2.1 PlantVillage dataset

**Figure 1:**
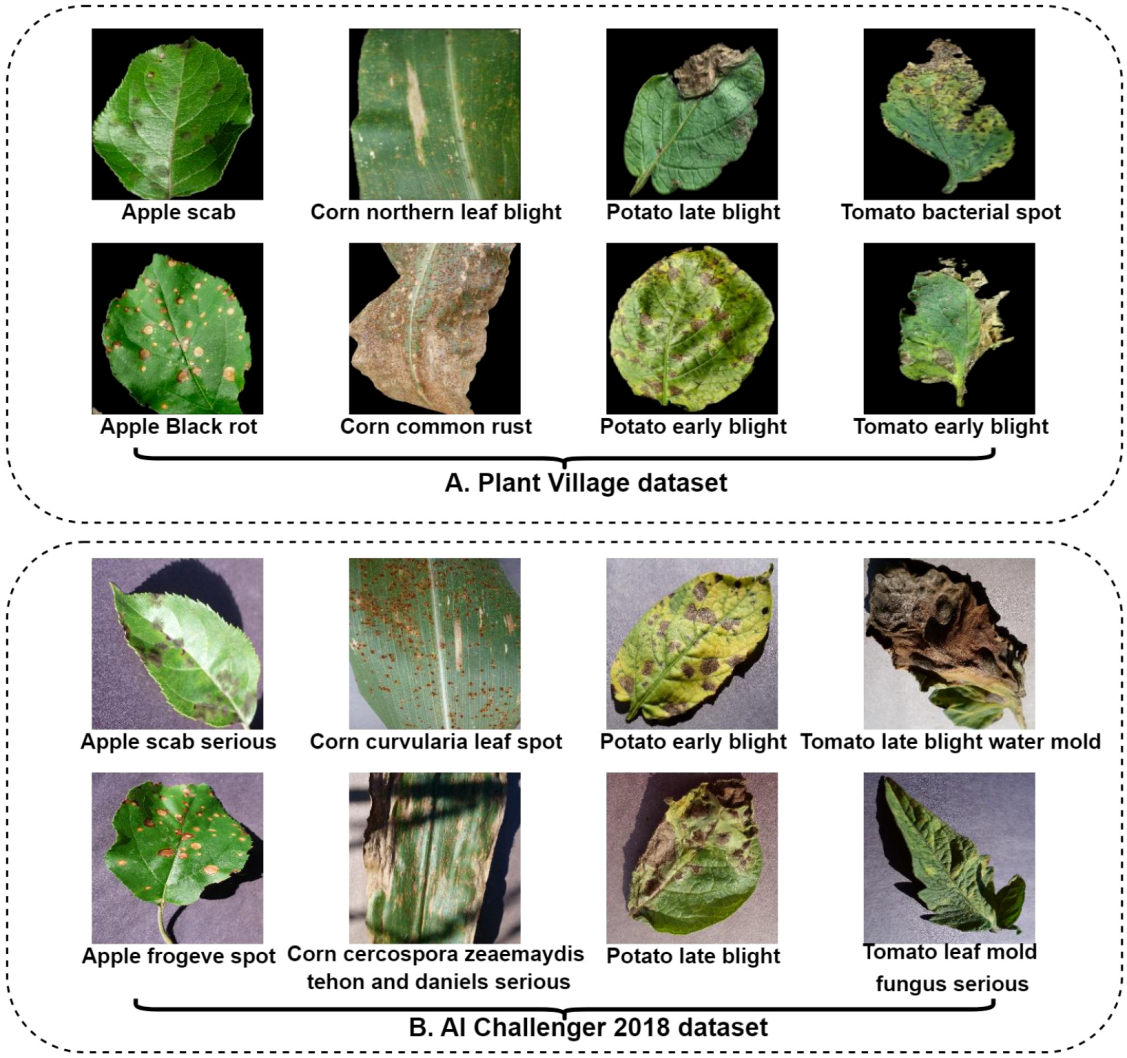
Sample images of diseased leaves from the PlantVillage dataset and the AI Challenger 2018 dataset.

In this study, we utilized the highly regarded PlantVillage dataset (Davi et al., 2015) (Hughes and Marcel, 2015), which includes 38 categories of plant leaf diseases and a total of 54,305 images. This dataset consists of 36,219 images in the training set, 9,036 images in the validation set, and 9,050 images in the test set, with example images shown in **Figure 1**.A. This dataset was chosen for its widespread use as a benchmark in plant disease recognition, providing a robust foundation for initial model validation and comparison with existing literature. Each image in this dataset has been rigorously validated and annotated by experienced plant pathologists, ensuring a high level of accuracy and reliability.

### 2.2 AI challenger 2018 dataset

To further validate the advancements and generalizability of our method, we also utilized the AI Challenger 2018 Dataset (Wu et al., 2019) (Wu et al., [[NoYear]]), which comprises 61 categories of plant leaf diseases at varying degrees of severity, totaling 36,075 images. This dataset includes 25,252 images in the training set, 6,289 images in the validation set, and 4,534 images in the test set, with example images displayed in **Figure 1**.B. Its highly imbalanced category labels make recognition inherently more challenging. This dataset serves as a critical test for model robustness under difficult conditions. In this study, we treated it as an ablation dataset and did not apply any augmentation techniques to specifically assess the model’s inherent performance on imbalanced data without external data manipulation.

### 2.3 Lightweight multi-path pruning method

**Figure 2:**
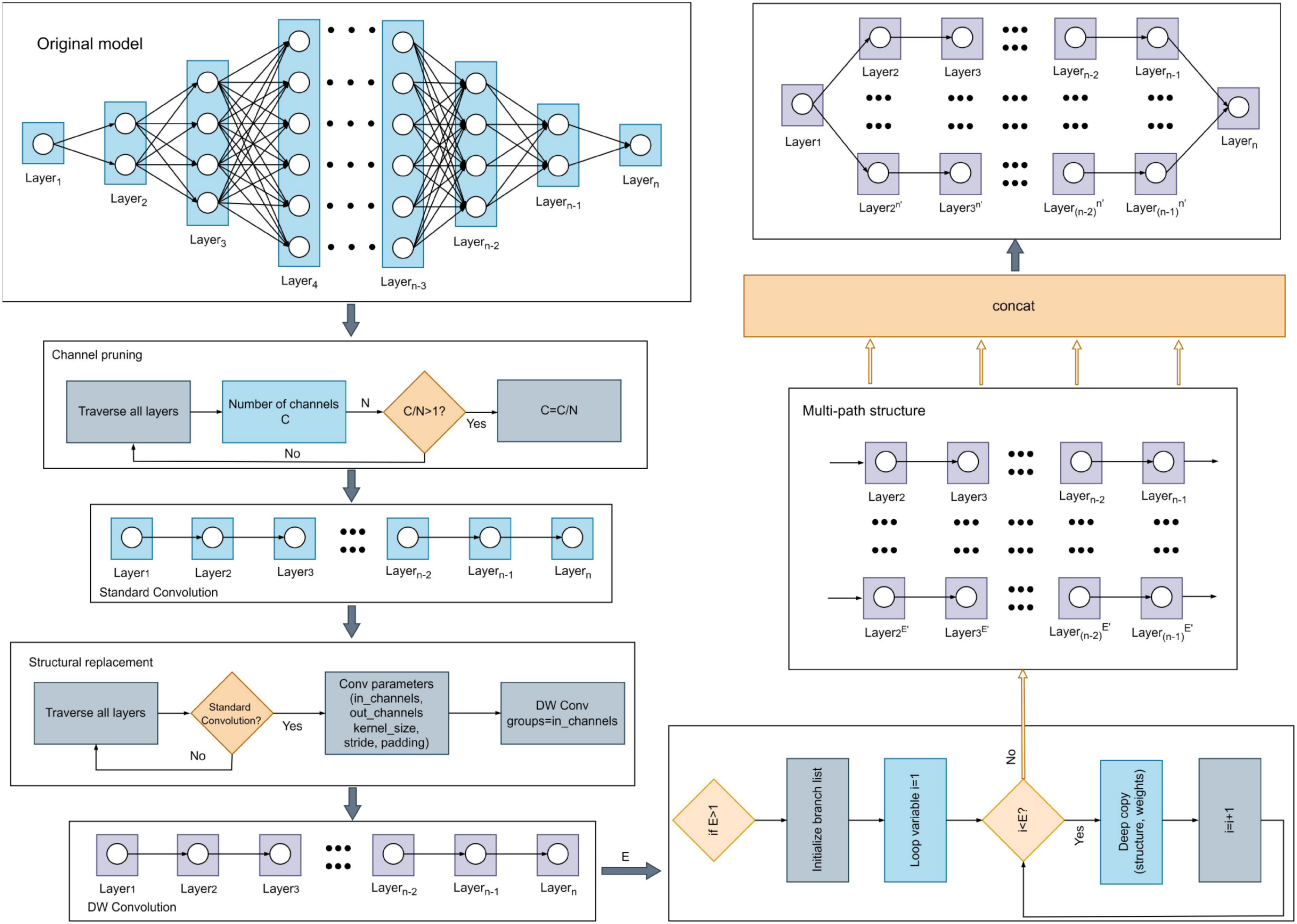
The overall architecture of lightweight multi-path pruning method.

We propose a novel Lightweight Multi-Path Pruning Method (LMP-PM), as illustrated in **Figure 2**. First, we design an original model, assuming the input feature map is represented by $X\in R^{C\times A\times B}$, with C indicating the number of input channels, and A and B representing the height and width of the input feature map, respectively. We define W as the weight matrix of the original convolutional layer; therefore, the output of the original model can be expressed by formula (1):

(1) $$Y_{original}=\sum_{i=1}^{C}\sum_{j=-\frac{K}{2}}^{\frac{K}{2}}\sum_{k=-\frac{K}{2}}^{\frac{K}{2}}W_{f,i,j,k}\cdot X_{i,a+j,b+k},$$

where f represents the index of the output channels, and $X_{i,a+j,b+k}$ is the input feature map. K denote the size of the convolutional kernel, with its center position located at ( $\frac{K}{2}$, $\frac{K}{2}$). Considering the symmetry of the convolutional kernel, indices from $-\frac{K}{2}$ to $\frac{K}{2}$ can cover the entire kernel. Next, we proceed to lightweight the original model by reducing the number of input feature map channels to $\frac{C}{N}$. N is the pruning parameter, and its size is determined based on the complexity of the original model. The specific steps for pruning are as follows: First, we iterate through all layers, sequentially extracting each standard convolutional layer from the original model. For the current convolutional layer, we retrieve its output channel count, denoted as C. Next, a custom pruning parameter, N, is introduced. The value of this parameter N is typically determined based on the original model’s complexity and the desired degree of lightweighting. Subsequently, we check if the current layer’s output channel count satisfies the pruning condition, specifically C/N > 1. If this condition is not met, the layer is not pruned, thereby preventing excessive compression that could lead to a drastic performance drop. If the pruning condition is met, the layer’s output channel count C is updated to C = C/N. This action, in turn, reduces the computational burden on this layer and subsequent layers. Finally, these steps are repeated until all convolutional layers have been traversed and their channel counts adjusted according to the pruning conditions. We define the new convolutional weights as W1, therefore, the output feature map can be expressed as formula (2):

(2) $$Y_{1}=\sum_{i=1}^{\frac{C}{N}}\sum_{j=-\frac{K}{2}}^{\frac{K}{2}}\sum_{k=-\frac{K}{2}}^{\frac{K}{2}}W_{1,f_{1},i,j,k}\cdot X_{i,a+j,b+k},$$

where the new convolutional weights $W_{1}\in R^{F_{1}\times \frac{C}{N}\times K\times K}$, $f_{1}$ serve as indices for the new output channels. Following this, we perform the next lightweight operation by replacing the standard convolution with depthwise (DW) convolution, as outlined in (Howard et al., 2017). Subsequently, the process entails re-traversing all layers within the channel-pruned model to determine whether the current layer is a standard convolutional layer. If so, its critical parameters—namely, in_channels, out_channels, kernel_size, stride, and padding—are extracted. These extracted parameters are then utilized to construct a depthwise convolutional layer. At this point, the input feature map, denoted as $Y_{1}$, processed to obtain formula (3):

(3) $$Y_{2}=\sum_{i=1}^{\frac{C}{N}}\sum_{j=-\frac{K}{2}}^{\frac{K}{2}}\sum_{k=-\frac{K}{2}}^{\frac{K}{2}}W_{d,i,j,k}\cdot Y_{1,i,a+j,b+k},$$

where the weights of the depthwise convolution are $W_{d}\in R^{\frac{C}{N}\times 1\times K\times K}$, where j and k serve as the indices for the convolution operation. Immediately following this, we apply pointwise convolution to $Y_{2}$, resulting in formula (4):

(4) $$Y_{3}=\sum_{f_{2}=1}^{F_{2}}\sum_{i=1}^{\frac{C}{N}}W_{p,f_{2},i}\cdot Y_{2,i,a,b}.$$

Then, to balance the performance loss associated with the two lightweight operations, we convert the single-path model into a multi-path model by controlling the path expansion factor E. The specific steps are as follows: First, we examine the value of the custom path expansion factor, E. If E ≤ 1, multi-path expansion is not performed; instead, the lightweight single-path model is utilized directly. However, if E > 1, an empty list is initialized to store the multiple model branches that will be subsequently created. A loop is then initiated, with the loop variable ‘i’ iterating from 1 to E. In each iteration, a deep copy of the structurally-transformed lightweight single-path model is performed. This implies copying not only the model’s architecture but also all of its current weights. Afterwards, the weights of all branches except the original one are randomly initialized. The duplicated model instance is then appended to the pre-initialized list of branches. Upon the loop’s completion, the number of paths is determined based on the specific task, resulting in formula (5):

(5) $$Y_{p_{E=1}}=Y_{p_{E=2}}=\cdot \cdot \cdot =Y_{p_{E=n-1}}=Y_{p_{E=n}}=\sum_{f_{2}=1}^{F_{2}}\sum_{i=1}^{\frac{C}{N}}W_{p,f_{2},i}\cdot (\sum_{i=1}^{\frac{C}{N}}\sum_{j=-\frac{K}{2}}^{\frac{K}{2}}\sum_{k=-\frac{K}{2}}^{\frac{K}{2}}W_{d,i,j,k}\cdot X_{i,a+j,b+k}).$$

Finally, we concatenate all the paths, after lightweight optimization, the final output results of the model are shown in formula (6):

(6) $$Y_{final}=Concat(Y_{p_{E}{=}_{1}},Y_{p_{E=2}},\cdot \cdot \cdot ,Y_{p_{E=n-1}},Y_{p_{E=n}};\dim=1).$$

### 2.4 OMNet

**Figure 3:**
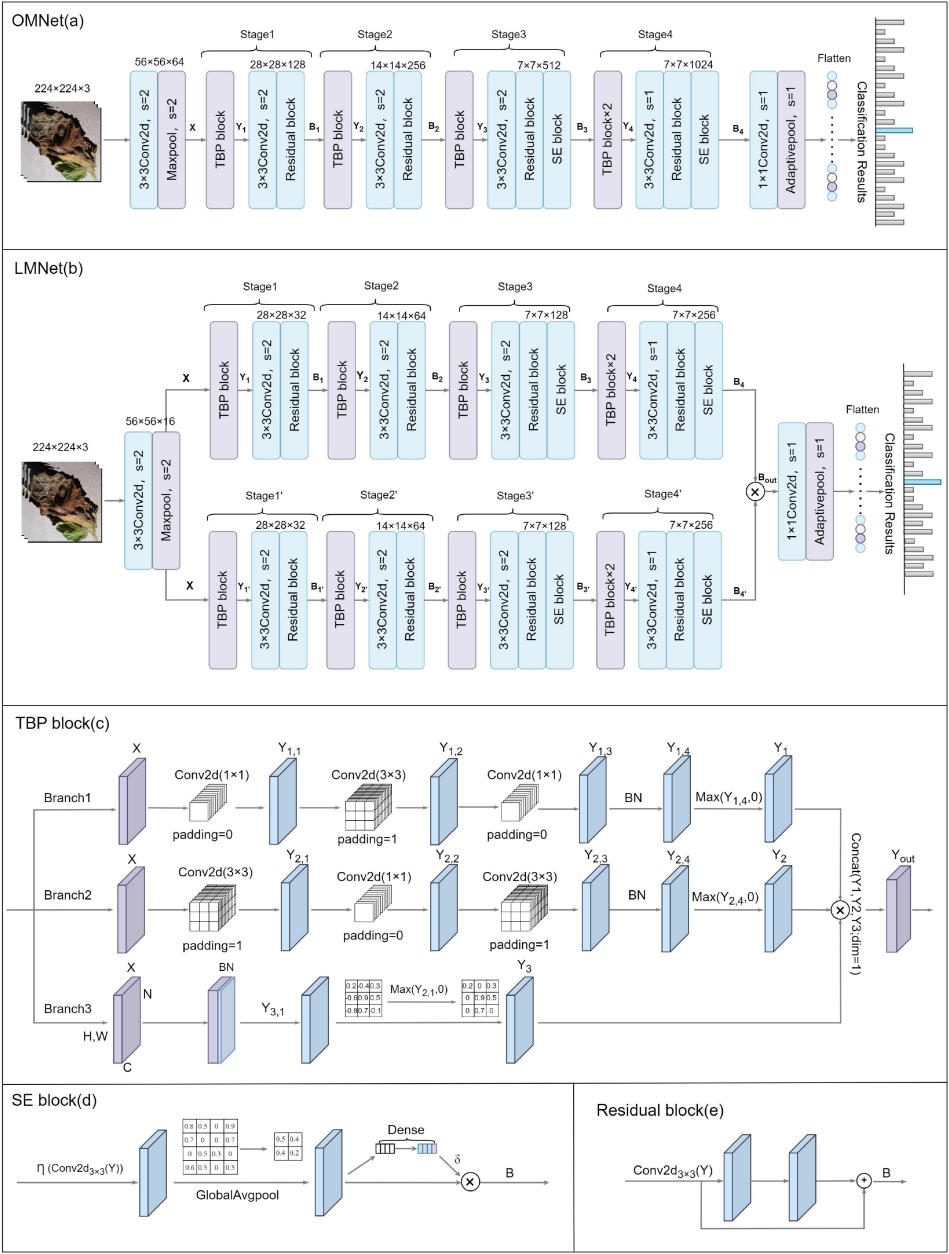
Model structure diagrams of ONET, LMNet, and TBP block.

We propose an original model (OMNet), whose structure is illustrated in **Figure 3A**. OMNet was designed to deliver high performance and high complexity, serving as the foundation for subsequent lightweight processing in LMP-PM. OMNet comprises fundamental components such as convolutional layers, pooling layers, activation functions, and normalization functions, while also integrating residual modules (He et al., 2016), SE modules (Hu et al., 2018), and TBP blocks. The overall design approach of OMNet involves first downsampling the input image twice, reducing the feature map size by four times while maintaining the number of channels to facilitate subsequent feature extraction. Next, these feature maps enter the main feature extraction structure, which is also the focus of the subsequent validation of LMP-PM. For ease of representation, we divide this portion of the structure into four stages. Let the input image for the first stage be represented as X. Here, we omit the calculations related to batch normalization (BN) and ReLU, and the output of the first stage can be expressed by formula (7) as:

(7) $$B_{1}=\eta_{1}(Conv2d_{3\times 3}(Y_{1}X)),$$

where, $Y_{1}X$ represents the TBP block operation in the first stage, while η denotes the residual module operation. The output of the second stage is similar to that of the first stage, as shown in equation (8):

(8) $$B_{2}=\eta_{2}(Conv2d_{3\times 3}(Y_{2}B_{1})).$$

Compared to the first two stages, the third stage includes an additional SE block. The output of the third stage can be expressed by formula (9):

(9) $$B_{3}=\delta (Dense(GlobalAvgpool(\eta_{3}(Conv2d_{3\times 3}(Y_{3}^{B_{2}})))))\cdot \eta_{3}(Conv2d_{3\times 3}(Y_{3}^{B_{2}})),$$

where, δ represents the Sigmoid activation function in the SE block, while Dense refers to the fully connected layer within the SE block. In the fourth stage, an additional TBP block is added compared to the third stage, and the other components remain similar. Output as shown in formula (10):

(10) B4=δ(Dense(GlobalAvgpool(η4(Conv2d3×3(Y4B3')))))·η4(Conv2d3×3(Y4B3')),

where, Y4B3' denotes the operations of the TBP block performed twice. Subsequently, the feature maps outputted from the fourth stage are passed through a 1×1 convolutional layer followed by an Adaptive Pooling layer. Finally, these are flattened and mapped to the output classes by a fully connected layer.

### 2.5 LMNet

LMNet is a lightweight model derived by applying our proposed Lightweight Multi-Path Pruning Method (LMP-PM) to OMNet, with its structure illustrated in **Figure 3B**. LMNet significantly reduces the model’s parameter count and computational complexity while maintaining performance superior to OMNet.

The lightweighting of LMNet is primarily achieved through two core strategies: global channel pruning and path expansion. Compared to OMNet, LMNet reduces the global channel count by one-fourth across the entire network. This implies that the channel dimensions of both convolutional and fully connected layers within the network are proportionally scaled down, leading to a substantial reduction in model parameters and FLOPs.

To compensate for potential performance degradation caused by channel pruning and to enhance the model’s feature extraction capabilities, OMNet’s main feature extraction component is expanded into two independent, parallel paths. Each path, in itself, constitutes a pruned and lightweighted OMNet main feature extraction structure. These two paths share the same internal structure, but their parallel processing enables the capture of richer and more diverse feature representations.

Specifically, LMNet’s feature extraction process unfolds as follows: The input image first passes through the same initial downsampling layer as OMNet. Subsequently, the resulting feature maps simultaneously feed into two independent, pruned OMNet main feature extraction structures. These two computational processes are analogous to OMNet’s main feature extraction process detailed in Section 2.4, but with proportionally pruned channel dimensions. The output feature maps from the two parallel paths are then concatenated dimensionally to form a broader feature representation. The final output can be expressed by formula (11):

(11) Bout=Concat(B4,B4';dim=1),

where, the computation processes for $B_{4}$ and B4' are similar to those described in Section 2.4. Subsequently, $B_{out}$ passes through a 1×1 convolutional layer and then an Adaptive Pooling layer before being flattened. Finally, it is mapped to the output classes by a fully connected layer.

### 2.6 TBP block

We designed a TBP block, and its specific structure is illustrated in **Figure 3C**. The input image simultaneously enters branches 1, 2, and 3, where corresponding feature extraction tasks are performed before the features are fused. This design facilitates the extraction of multi-scale complex feature information, thereby enhancing the performance of OMNet. Let X denote the input with dimensions (N,C,H,W), where N is the batch size, C is the number of input channels, and H and W represent the height and width of the input feature map, respectively. The output is denoted as Y. The feature map input to branch 1 first passes through a 1×1 convolutional layer, the output is as shown in formula (12):

(12) $$Y_{1,1}=Conv2d_{1\times 1}(X;C,1,S),$$

where, S represents the stride, which is set to 1 by default. The feature map then passes through a second convolutional layer, as shown in formula (13):

(13) $$Y_{1,2}=Conv2d_{3\times 3}(Y_{1,1};C,3,S,Padding=1).$$

After passing through a third convolutional layer, we obtain formula (14):

(14) $$Y_{1,3}=Conv2d_{1\times 1}(Y_{1,2};C,1,S),$$

where $Y_{1,3}$ undergoes batch normalization, resulting in formula (15):

(15) $$Y_{1,4}=\frac{Y_{1,3}-\mu }{\sqrt{\sigma^{2}+\epsilon }}\cdot \gamma +\beta ,$$

where μ and $\sigma^{2}$ represent the mean and variance of the input, γ and β are the learnable parameters, and ϵ is a small constant to prevent division by zero. Ultimately, the activation mapping produces the result as shown in formula (16):

(16) $$Y_{1}=Max(\frac{Y_{1,3}-\mu }{\sqrt{\sigma^{2}+\epsilon }}\cdot \gamma +\beta ,0).$$

Similarly, the output of the second branch can be expressed as formula (17):

(17) $$Y_{2}=Max(\frac{Y_{2,3}-\mu }{\sqrt{\sigma^{2}+\epsilon }}\cdot \gamma +\beta ,0),$$

the output of the third branch is shown in Formula (18):

(18) $$Y_{3}=Max(\frac{Y_{3,3}-\mu }{\sqrt{\sigma^{2}+\epsilon }}\cdot \gamma +\beta ,0).$$

Finally, the outputs of the three branches are merged through a concatenation operation as shown in Formula (19):

(19) $$Y_{out}=Concat(Y_{1},Y_{2},Y_{3};\dim=1),$$

After substituting the numerical values, we obtain the comprehensive output formula of the TBP block as shown in Formula (20):

(20) $$Y_{out}=Concat(Max(\frac{Y_{1,3}-\mu_{1}}{\sqrt{\sigma_{1}2+\epsilon }}\cdot \gamma_{1}+\beta_{1},0),Max(\frac{Y_{2,3}-\mu_{2}}{\sqrt{\sigma_{2}2+\epsilon }}\cdot \gamma_{2}+\beta_{2},0),Max(\frac{X-\mu_{3}}{\sqrt{\sigma_{3}2+\epsilon }}\cdot \gamma_{3}+\beta_{3},0);\dim=1)$$

## 3 Results

### 3.1 Experimental design

Our experimental conditions are presented in **Table 1**. All ablation experiments were conducted under the following conditions: an initial learning rate of 1×10^-4^, 100 iterations, the AdamW optimizer (Loshchilov and Hutter, 2017), a weight decay of 5×10^-2^, and a cross-entropy loss function. To ensure the comparison reflects the model’s original performance, no pre-training was applied in any of the experiments. In order to validate the effectiveness of LMP-PM and to demonstrate that LMNet possesses strong performance and generalization capabilities, we conducted four relevant experiments. These included verifying the effectiveness of LMP-PM using two excellent open-source agricultural image datasets, testing the efficacy of the TBP block, assessing the lightweight high-performance attributes of LMNet, and finally, performing a visualization validation. To enhance model robustness and prevent overfitting, standard data augmentation techniques were applied to the training data for the PlantVillage dataset. These included Random resized crop (224x224), Random rotation ([-10°, 10°]), and Random horizontal flip (0.5 probability). Images were then normalized to a [0.0, 1.0] range. For the AI Challenger 2018 dataset, no augmentation techniques were applied, as stated in Section 2.2, to specifically evaluate the model’s performance on its inherent data distribution and imbalance.

**Table 1:** Experimental conditions.

Device	Version
GPU	ASUS RTX 4070 Ti
CPU	Intel i5-12490F
Memory	G.Skill 32 GB RAM
Frame	PyTorch 2.0.1
Tool	Python 3.9.13
GPU accelerator 1	CUDA 11.8
GPU accelerator 2	CUDNN 8.9.4.25
Learning rate	1×10^-4^
weight decay	5×10^-2^
Optimizer	AdamW
Epoch	100
Random resized crop	224×224
Random rotation	[-10°, 10°]
Random horizontal flip	0.5
Normalization	[0.0, 1.0]

### 3.2 Evaluation indicators

We employed several commonly used evaluation metrics to assess the performance of LMNet, including accuracy, precision, recall, and the F1 score, as defined by formulas (21-24). We used the number of parameters and FLOPs as indicators of the model’s lightweight characteristics. Additionally, we utilized precision-recall (PR) curves, ROC curves, and confusion matrices to further evaluate LMNet’s classification performance.

(21) $$Accuracy=\frac{\left(TP+TN\right)}{\left(TP+TN+FP+FN\right)},$$

(22) $$Recall=\frac{TP}{\left(TP+FN\right)},$$

(23) $$Precision=\frac{TP}{\left(TP+FP\right)},$$

(24) $$F1-score=\frac{2\times (Precision\times Recall)}{\left(Precision+Recall\right)},$$

where TP denotes the number of true-positive samples, FP denotes the number of false-positive samples, FN denotes the number of false-negative samples, and TN denotes the number of true-negative samples.

### 3.3 Verification of the effectiveness of the LMP-PM

To validate the effectiveness of LMP-PM, we designed two sets of experiments, including verification of the lightweight capability of model pruning and assessment of the accuracy enhancement capability through path expansion, thereby comprehensively evaluating the performance of LMP-PM.

#### 3.3.1 Verification of the effectiveness of model pruning

To verify the impact of model pruning on performance, we compared various performance metrics between the original model (N = 1, E = 1) and the pruned model, with experimental results presented in **Table 2**. As the pruning parameters increased, the parameters and FLOPs of OMNet significantly decreased. When N = 16, further pruning of OMNet resulted in the first convolution having fewer than three output channels, rendering it unable to extract features from color images. Therefore, N = 16 represents the minimum pruned model, with OMNet’s parameters reduced from 99,799,144 to 279,884, and FLOPs decreased from 10,371,351,552 to 14,956,000, representing reductions of approximately 358 times and 693 times, respectively. It is noteworthy that while the size of OMNet was greatly reduced at this stage, excessive pruning led to a significant decline in model accuracy. On the Plant Village dataset, the model (N = 16, E = 1) exhibited a 6.27% decrease in Test accuracy compared to OMNet, alongside reductions of 7.76% in Precision, 7.59% in F1-score, and 7.41% in Recall. A similar phenomenon was observed on the AI Challenger 2018 dataset, where the model (N = 16, E = 1) showed a 5.47% decrease in Test accuracy compared to OMNet, with Precision reduced by 10.45%, F1-score by 12.41%, and Recall by 12.91%.

**Table 2:** Comparison of model performance after pruning (due to the limited lightweight effect of OMNet when the pruning parameter N = 2, we have excluded the models from this group).

Method	Plant village dataset				AI challenger 2018 dataset				Parameters	FLOPs
	Test-acc	Precision	F1-score	Recall	Test-acc	Precision	F1-score	Recall		
N=1, E = 1	98.65%	98.20%	97.94%	97.79%	85.36%	82.16%	81.04%	80.67%	99799144	10371351552
N=4, E = 1	98.21%	97.69%	97.32%	97.20%	84.98%	79.43%	78.47%	78.20%	3353720	200291712
N=8, E = 1	95.80%	94.16%	94.32%	94.75%	83.79%	78.58%	76.43%	76.20%	1102640	53515968
N=16, E = 1	92.38%	90.44%	90.35%	90.38%	79.89%	71.71%	68.63%	67.76%	279884	14956000

**Figure 4:**
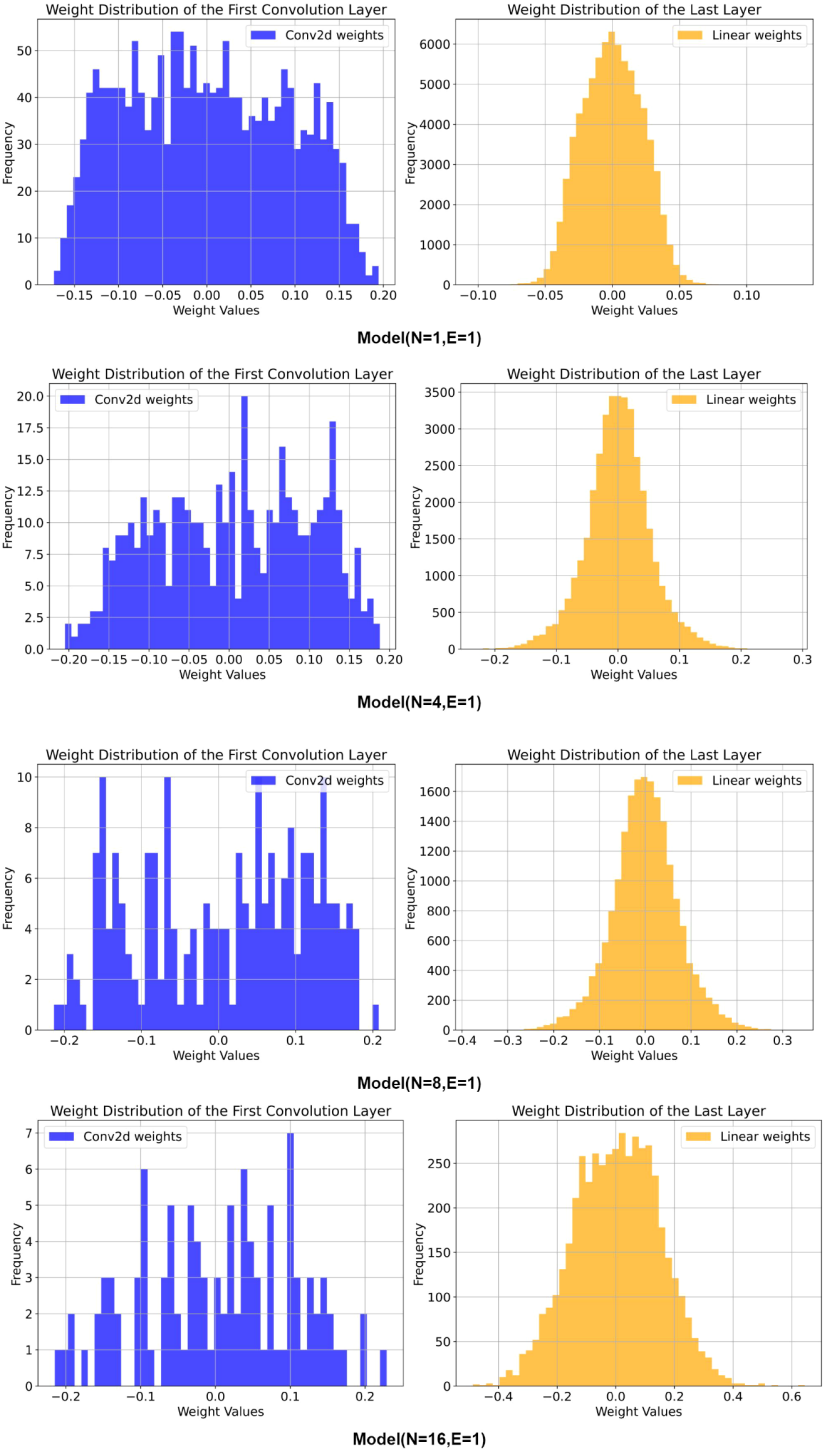
Weight distribution of the model before and after pruning.

**Figure 4** details the weight distribution of the first convolutional layer and the final fully connected layer under different pruning parameters. The frequency of weight values is represented on the left vertical axis, while the weight values themselves are displayed on the horizontal axis. From the figure, it is evident that the frequency in the final fully connected layer is significantly higher than that in the first convolutional layer, indicating that the fully connected layer has a greater impact on the model’s decisions. In contrast, the first convolutional layer primarily extracts shallow features, which have a relatively smaller influence on decision-making. The model with N = 1 and E = 1 exhibits the highest frequency in both the first convolutional layer and the last fully connected layer. For the other three models, the frequency gradually decreases as the pruning parameters increase, highlighting an improvement in parameter utilization. The first three models display similar weight distribution patterns in the final fully connected layer, suggesting that during the initial pruning stages, the model successfully eliminated many unimportant connections or neurons. Although the remaining weights are fewer in number, they are more concentrated, allowing the pruned models to maintain good decision-making capabilities. However, the third pruning stage resulted in a significant decline in the model’s decision-making ability, as the remaining weights could not be effectively concentrated. Notably, the first convolutional layer demonstrated strong sparsity at E = 16, with considerable differences in height among the values, indicating substantial variation among the remaining weights. This suggests that certain important features or connections are critical to the model, while others are deemed extraneous. While this enhances parameter utilization, it may also lead to a certain degree of information loss and variations in model performance.

#### 3.3.2 Verification of path expansion effectiveness

To address the significant performance degradation caused by model pruning, we conducted path expansion operations. Due to hardware constraints, we only explored five models with different path expansion ratios under varying pruning parameters. The results are presented in **Tables 3**–**5**.

**Table 3:** Comparison of models with different path expansion ratios when pruning parameter N = 4 (Bold font indicates the optimal model data).

Method	Plant village dataset				AI challenger 2018 dataset				parameters	FLOPs
	Test-acc	Precision	F1-score	Recall	Test-acc	Precision	F1-score	Recall		
N=1, E = 1	98.65%	98.20%	97.94%	97.79%	85.36%	82.16%	81.04%	80.67%	99799144	10371351552
N=4, E = 1	98.21%	97.69%	97.32%	97.20%	84.98%	79.43%	78.47%	78.20%	3353720	200291712
N=4, E = 2	**99.23%**	**98.97%**	**98.94%**	**98.91%**	**87.27%**	**83.72%**	**82.91%**	**83.06%**	5679944	394008320
N=4, E = 3	98.86%	98.71%	98.51%	98.35%	85.75%	79.47%	78.47%	78.16%	8519192	588302976
N=4, E = 4	98.96%	98.67%	98.56%	98.49%	86.52%	81.87%	81.80%	82.25%	11358440	782597632
N=4, E = 5	98.95%	98.59%	98.61%	98.65%	85.80%	80.79%	79.46%	78.82%	14197688	976892288

**Table 4:** Comparison of models with different path expansion ratios when pruning parameter N = 8.

Method	Plant village dataset				AI challenger 2018 dataset				Parameters	FLOPs
	Test-acc	Precision	F1-score	Recall	Test-acc	Precision	F1-score	Recall		
N=1, E = 1	98.65%	98.20%	97.94%	97.79%	85.36%	82.16%	81.04%	80.67%	99799144	10371351552
N=8, E = 1	95.80%	94.16%	94.32%	94.75%	83.79%	78.58%	76.43%	76.20%	1102640	53515968
N=8, E = 2	98.48%	97.76%	97.93%	98.15%	**86.66%**	**82.95%**	**81.74%**	**81.54%**	1691032	103744384
N=8, E = 3	97.16%	96.09%	96.12%	96.24%	84.85%	79.44%	78.18%	77.82%	2535936	154261824
N=8, E = 4	98.65%	**98.35%**	98.27%	98.22%	86.35%	82.36%	81.53%	81.39%	3380840	204779264
N=8, E = 5	**98.74%**	98.16%	**98.31%**	**98.48%**	85.75%	82.10%	81.25%	80.94%	4225744	255296704

Bold font indicates the optimal model data.

**Table 5:** Comparison of models with different path expansion ratios when pruning parameter N = 16.

Method	Plant village dataset				AI challenger 2018 dataset				Parameters	FLOPs
	Test-acc	Precision	F1-score	Recall	Test-acc	Precision	F1-score	Recall		
N=1, E = 1	98.65%	98.20%	97.94%	97.79%	85.36%	82.16%	81.04%	80.67%	99799144	10371351552
N=16, E = 1	92.38%	90.44%	90.35%	90.38%	79.89%	71.71%	68.63%	67.76%	279884	14956000
N=16, E = 2	97.30%	96.18%	96.30%	96.53%	83.35%	77.85%	75.40%	75.02%	558656	28557248
N=16, E = 3	96.51%	95.12%	95.41%	95.89%	82.49%	76.39%	73.26%	72.76%	837428	42158496
N=16, E = 4	**97.48%**	**96.84%**	**96.89%**	**96.99%**	**84.80%**	**79.79%**	**78.64%**	**78.48%**	1116200	55759744
N=16, E = 5	97.31%	96.48%	96.41%	96.44%	82.31%	76.15%	73.39%	72.79%	1394972	69360992

Bold font indicates the optimal model data.

**Table 3** provides a detailed record of various model performance metrics when N = 4, under five different path expansion ratios. Compared to OMNet (E = 1, N = 1), all models, except for the model without path expansion (E = 4, N = 1), exhibited performance that surpassed that of OMNet, with a significant reduction in both parameters and FLOPs. Among the five models under the condition of N = 4, the model with N = 4 and E = 2 demonstrated the best performance. On the Plant Village dataset, this model achieved a Test accuracy improvement of 0.58% over OMNet, with Precision increasing by 0.77%, F1-score by 1.00%, and Recall by 1.12%. On the AI Challenger 2018 dataset, the model (N = 4, E = 2) outperformed OMNet with a Test accuracy improvement of 1.91%, Precision rising by 1.56%, F1-score by 1.87%, and Recall by 2.39%. Notably, the parameters and FLOPs for the model (N = 4, E = 2) were only 5.69% and 3.80% of those for OMNet, respectively.

**Table 4** presents the model performance metrics under five different path expansion ratios when N = 8. Compared to N = 4, the model sizes continued to decrease, and some models after path expansion still slightly outperformed OMNet, such as the models with N = 4 and N = 5. Notably, the model (E = 8, N = 5) achieved the best performance on the Plant Village dataset, with a Test accuracy improvement of 0.09% over OMNet, an increase in F1-score of 0.37%, and a Recall enhancement of 0.69%. The parameters and FLOPs for this model were only 4.24% and 2.46% of those for OMNet, respectively. Meanwhile, the model (E = 8, N = 2) exhibited the best performance on the AI Challenger 2018 dataset, achieving a Test accuracy improvement of 1.30% over OMNet, with Precision rising by 0.79%, F1-score by 0.70%, and Recall by 0.87%. The parameters and FLOPs for this model were merely 1.69% and 0.998% of those for OMNet.

**Figure 5:**
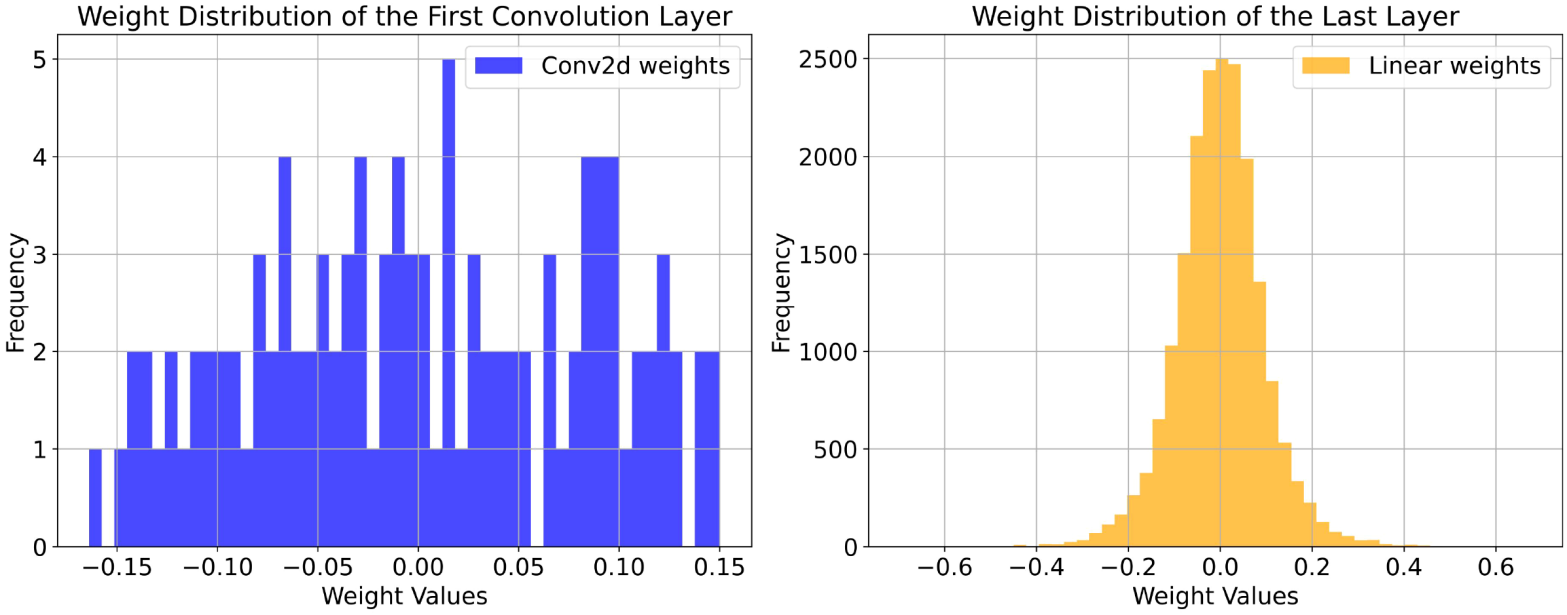
Weight distribution of the model (N = 16, E = 4).

**Table 5** presents the performance metrics of five models obtained through path expansion on the minimal model when N = 16. At this stage, the model complexity has reached its lowest, resulting in a significant decrease in model accuracy. However, applying path expansion to the minimal model still yielded substantial performance improvements, with the model (N = 16, E = 4) achieving the best results. Compared to the model (N = 16, E = 1), the Test accuracy on the Plant Village dataset improved by 5.10%, Precision increased by 6.10%, F1-score rose by 6.54%, and Recall enhanced by 6.61%. On the AI Challenger 2018 dataset, the Test accuracy increased by 4.91%, Precision improved by 8.08%, F1-score rose by 10.01%, and Recall increased by 10.72%. Although the accuracy of the model (N = 16, E = 4) does not surpass that of OMNet, its Test accuracy on the Plant Village dataset is approximately 98.81% of OMNet’s, and on the AI Challenger 2018 dataset, it is about 99.34% of OMNet’s. Notably, the parameters of the model (N = 16, E = 4) constitute only 1.12% of those for OMNet, while its FLOPs are merely 0.537% of OMNet’s. The weight distribution of the model (N = 16, E = 4) is illustrated in **Figure 5**, which shows a more concentrated weight distribution in the final fully connected layer compared to the model (N = 16, E = 1) shown in **Figure 4**. This phenomenon indicates that path expansion is effective in mitigating the performance degradation caused by excessive pruning.

**Figure 6:**
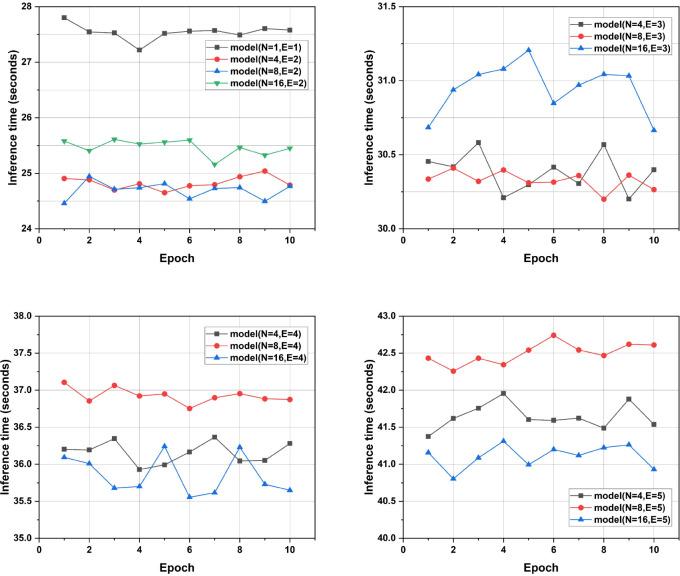
Inference time of the model on the plant village test dataset.

We collected inference time data for OMNet and path expansion models on the same device and during the same time period, recording ten data points for each model, as shown in **Figure 6**. The results indicate that the path expansion ratio significantly impacts the model’s inference time. When the path expansion ratio is the same, the inference time does not decrease with the increased degree of pruning. This may be due to the additional reconstruction or adjustments required by model pruning, which could increase inference time. Furthermore, when varying the path expansion ratios, the inference time noticeably increases with higher path expansion ratios. At E = 3 and E = 4, the inference time of the models has already surpassed that of OMNet. This observation suggests that when utilizing LMP-PM, it is essential to consider the increase in inference time associated with higher path expansion ratios.

**Figure 7:**
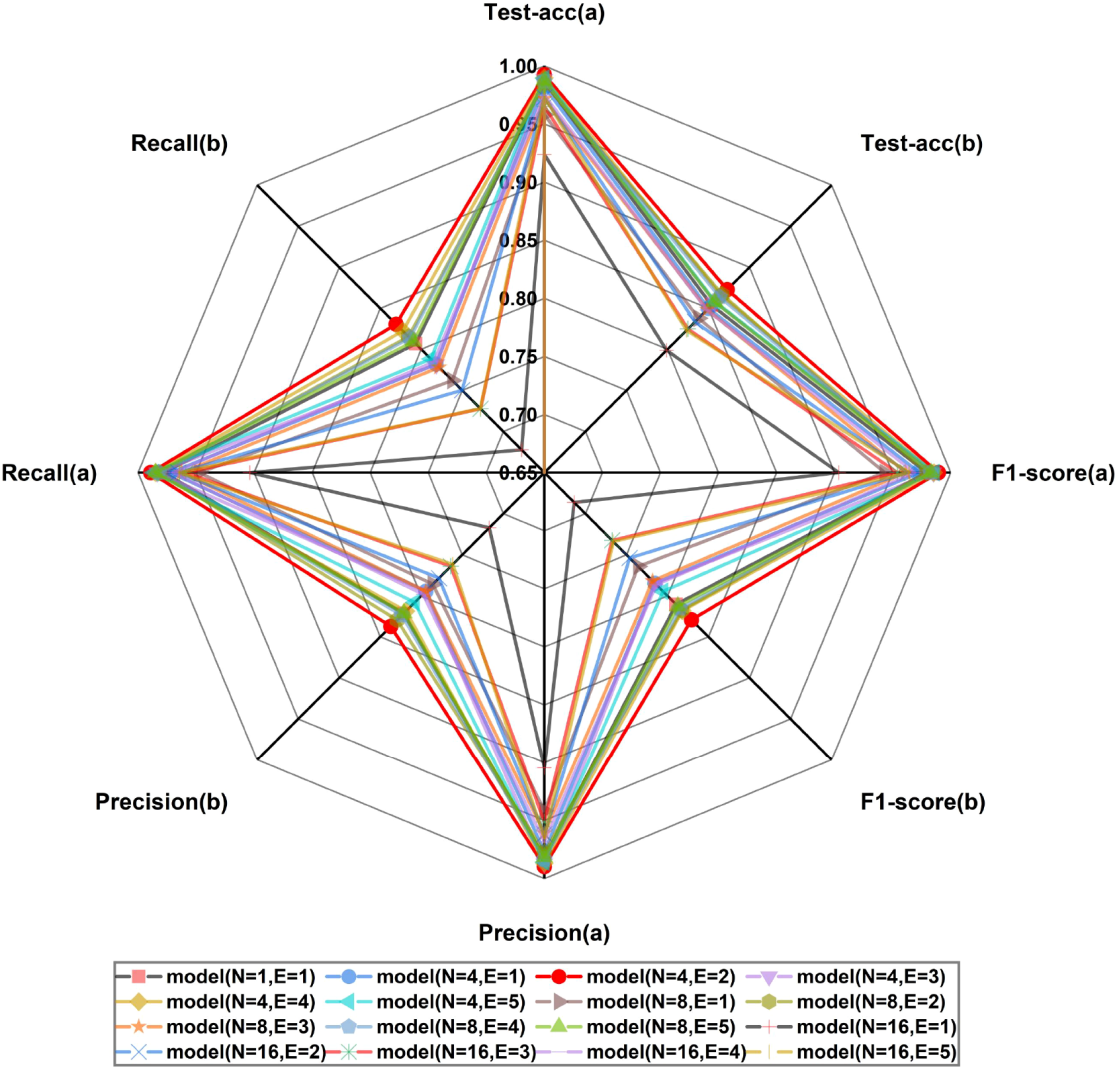
Accuracy evaluation metrics of the pruned model. **(a)** Results on the plant village dataset. **(b)** Results on the AI challenger 2018 dataset.

The experimental results indicate that pruning operations on OMNet can effectively reduce the model’s complexity, though this often comes at the cost of model performance. However, the path expansion operation within LMP-PM successfully alleviates this performance decline and can even enhance the performance of OMNet, demonstrating the effectiveness of LMP-PM. In practical applications of LMP-PM, users can select the most appropriate values of N and E based on specific task requirements to achieve optimal lightweight benefits. The accuracy metrics of all models derived from pruning OMNet with LMP-PM are illustrated in **Figure 7**, where the model with N = 4 and E = 2 exhibits the best performance while also maintaining a shorter inference time than OMNet. We have designated this model as LMNet. Further experiments will be conducted to validate the high-performance characteristics of LMNet.

#### 3.3.3 LMP-PM generalization validation

To further validate the generalizability of LMP-PM, we used ResNet101 as the baseline model and pruned it using the LMP-PM method, obtaining multiple models. The experimental results on the Plant Village Dataset and AI Challenger 2018 Dataset are presented in **Table 6**. When N = 4 and E = 4, the pruned model achieved the best performance, with test accuracy on both datasets higher than that of the original Resnet101, and a substantial reduction in the number of parameters. This further demonstrates the effectiveness of LMP-PM.

**Table 6:** Results of ResNet101 pruning using LMP-PM.

Method	Plant village dataset	AI challenger 2018 dataset	Parameters
	Test-acc	Test-acc	
ResNet101	98.54%	86.30%	42.63 M
N=1, E = 1	98.32%	86.08%	23.72 M
N=2, E = 1	98.19%	85.16%	6.01 M
N=2, E = 4	98.63%	86.76%	24.02 M
N=4, E = 1	97.77%	83.13%	1.54 M
N=4, E = 4	**99.12%**	**86.83%**	6.16 M
N=8, E = 1	96.82%	82.75%	0.40 M
N=8, E = 2	98.04%	85.81%	0.81 M
N=16, E = 1	93.97%	81.36%	0.11 M
N=16, E = 4	97.62%	85.01%	0.44 M

Bold font indicates the optimal model data.

### 3.4 Verification of the effectiveness of the TBP block

**Figure 8:**
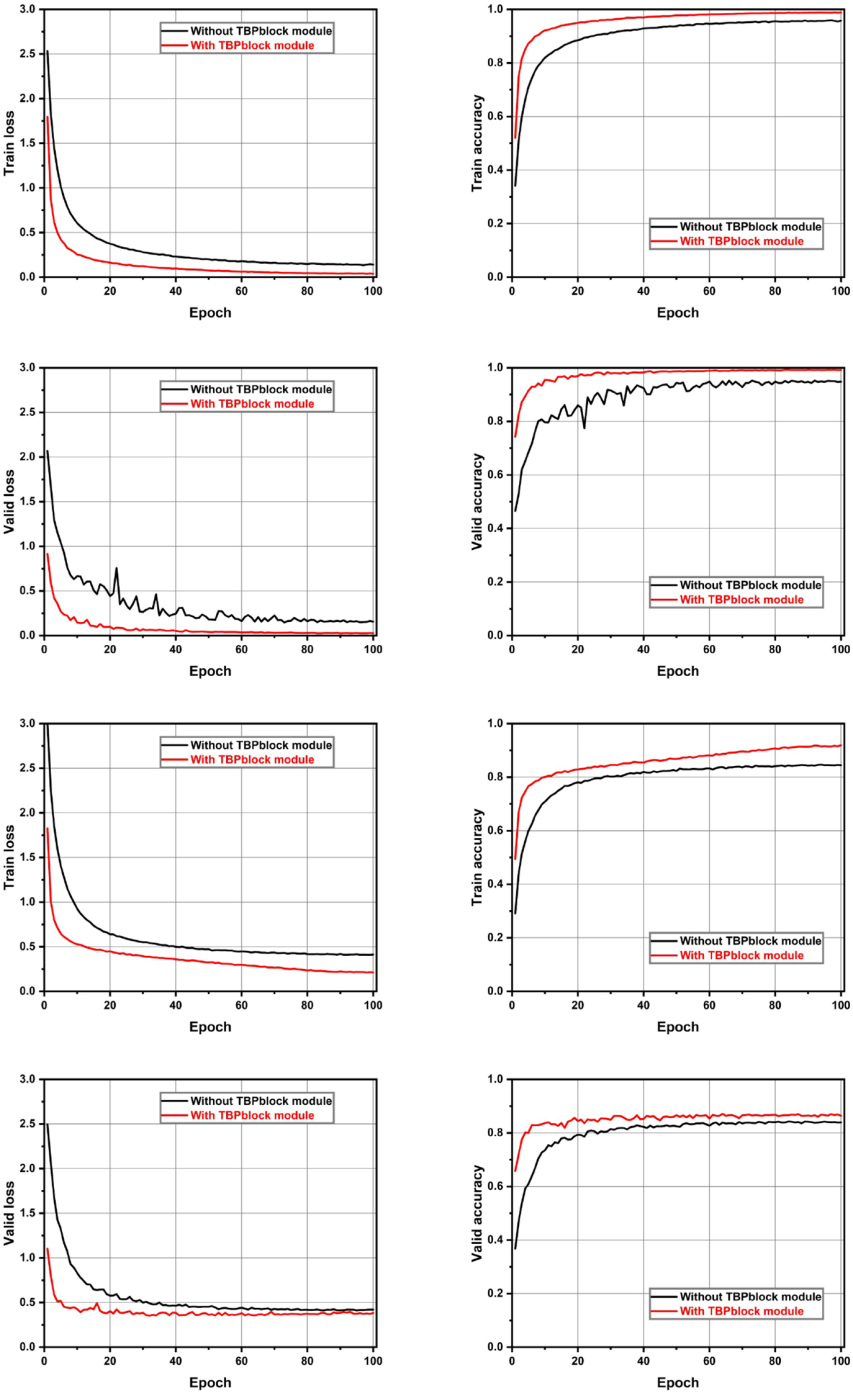
Comparison of the training processes for models with and without the TBP block. Up: Results on the Plant Village dataset. Down: Results on the AI Challenger 2018 dataset.

To validate the effectiveness of the TBP block, we used LMNet as the baseline model to compare the performance of models with and without the TBP block. The results are presented in **Table 7**. The model with the TBP block outperformed the model without it on both test sets, achieving a significant increase of 3.88% on the Plant Village dataset and 3.48% on the AI 2018 Challenger dataset. Additionally, all other performance evaluation metrics for the model with the TBP block also surpassed those of the model without it. The accuracy and loss trends during training on the training and validation sets for both models are illustrated in **Figure 8**. From the figure, it can be observed that the model with the TBP block demonstrates superior convergence speed and performance on both datasets. Notably, in the validation set of the Plant Village dataset, the stability of convergence with the TBP block is significantly better than that without it, effectively proving the validity of the TBP block.

**Table 7:** Comparison of the performance between models with and without the TBP block.

Method	Plant village dataset				AI challenger 2018 dataset			
	Test-acc	Precision	F1-score	Recall	Test-acc	Precision	F1-score	Recall
Without TBP block module	95.35%	93.88%	93.91%	94.26%	83.79%	77.95%	76.09%	75.30%
**With TBP block module**	**99.23%**	**98.97%**	**98.94%**	**98.91%**	**87.27%**	**83.72%**	**82.91%**	**83.06%**

Bold font indicates the optimal model data.

### 3.5 Comparison of LMNet with other outstanding models

**Figure 9:**
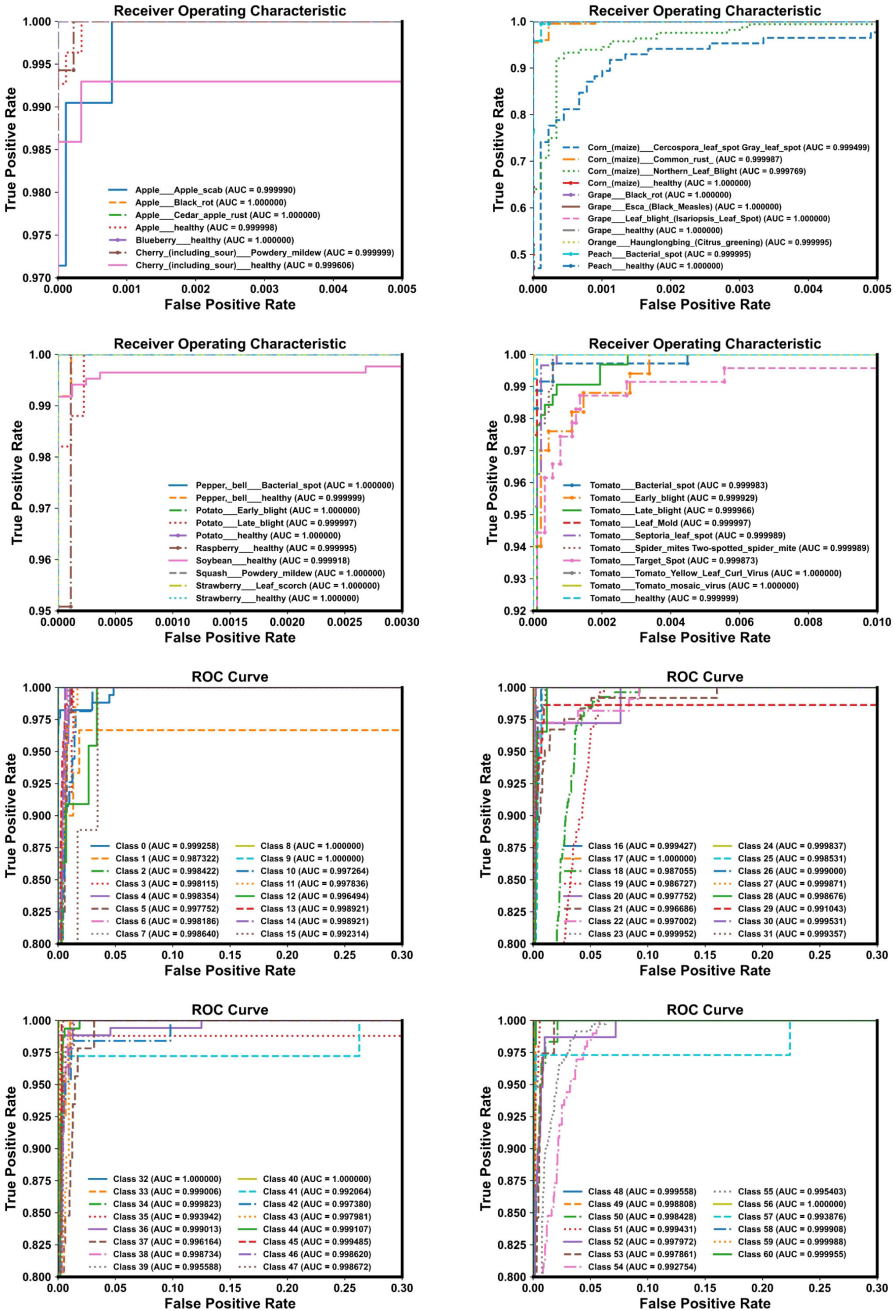
ROC curve diagram. Up: results on the plant village dataset. Down: Results on the AI challenger 2018 dataset.

To validate the performance of LMNet, we compared it with six other high-performing models. Among these, ConvNext served as the benchmark model due to its normal complexity and high performance, while the other five models are lightweight models with parameter counts similar to LMNet. The results are presented in **Table 8**. The experimental results indicate that LMNet achieved a Test accuracy on the Plant Village dataset that is 0.62% higher than the second-ranked Shufflenet_v2_x2_0. Additionally, its Precision is 0.97% higher, F1-score is 0.91% higher, and Recall is 0.82% higher. Notably, LMNet’s Test accuracy surpasses that of the benchmark model Convnext_tiny by 1.12%, while having only one-fifth of the parameters. On the AI 2018 Challenger dataset, LMNet continued to deliver the best performance, exceeding the second-ranked Repvit_m0_9 by 0.92% in Test accuracy, achieving 0.75% higher Precision, 1.38% higher F1-score, and 1.64% higher Recall. Furthermore, LMNet’s Test accuracy also outperformed the benchmark model Convnext_tiny by 5.31%. The changes in Accuracy and loss during training for these seven models on the training set and validation set are shown in **Figure 9**. From the figure, it is evident that LMNet exhibits superior convergence speed, stability, and overall performance compared to the other models, providing strong evidence for its outstanding capabilities.

**Table 8:** Performance comparison of LMNet with other models (no pre-training).

Method	Plant village dataset				AI challenger 2018 dataset				Parameters
	Test-acc	Precision	F1-score	Recall	Test-acc	Precision	F1-score	Recall	
ConvNeXt_tiny (Liu et al., 2022)	98.11%	97.75%	97.42%	97.15%	81.96%	74.74%	73.62%	73.35%	28589128
RegNetX_400MF (Szegedy et al., 2015)	97.06%	95.76%	96.00%	96.32%	85.29%	79.40%	78.31%	77.91%	5157512
Shufflenet_v2_x2_0 (Ma et al., 2018)	98.61%	98.00%	98.03%	98.09%	84.14%	78.30%	77.50%	77.40%	7393996
Mobilenet_v3_large (Howard et al., 2019)	97.45%	96.83%	96.68%	96.65%	86.13%	81.72%	80.65%	80.27%	5483032
DemoNet_d12_w256_sum (Ma et al., 2024)	94.69%	92.85%	93.19%	93.68%	82.40%	76.14%	74.80%	74.37%	7716584
Repvit_m0_9 (Wang A. et al., 2024)	98.39%	97.66%	97.81%	98.00%	86.35%	82.97%	81.53%	81.42%	5103560
EfficientNetB0 (Tan and Le, 2019)	98.57%	98.24%	98.09%	98.11%	84.45%	78.96%	78.20%	78.07%	8423848
GhostNet_3.0x (Han et al., 2020)	97.81%	97.26%	97.34%	97.28%	84.60%	80.07%	79.01%	78.99%	8469280
PVT_Tiny (Wang et al., 2021)	96.89%	96.25%	96.59%	96.41%	85.53%	80.16%	79.10%	79.27%	12900072
EdgeNeXt_Small (Maaz et al., 2022)	98.68%	98.00%	98.35%	98.16%	81.78%	78.16%	74.28%	74.85%	7463368
**LMNet**	**99.23%**	**98.97%**	**98.94%**	**98.91%**	**87.27%**	**83.72%**	**82.91%**	**83.06%**	5679944

Bold font indicates the optimal model data.

All comparative models in **Table 9** utilized pre-trained weights. On the Plant Village dataset, LMNet, with only 5.67M parameters, achieved a test accuracy of 99.23%, a score that even surpassed the pre-trained ConvNeXt_tiny (99.11%), despite the latter having a significantly larger parameter count of 28.6M. This comparison strongly demonstrates LMNet’s remarkable advantages in parameter efficiency and performance. Furthermore, on the AI Challenger 2018 dataset, LMNet achieved a test accuracy of 87.27%, ranking first among all models that used pre-trained weights. Moreover, LMNet also exhibited the best performance across key metrics such as Test-acc, Precision, F1-score, and Recall on this dataset. These results conclusively prove that LMNet can demonstrate exceptional generalization ability and leading performance, even without relying on pre-training.

**Table 9:** Performance comparison of LMNet with other outstanding models (pre-training).

Method	Plant village dataset				AI challenger 2018 dataset				Parameters
	Test-acc	Precision	F1-score	Recall	Test-acc	Precision	F1-score	Recall	
ConvNeXt_tiny (Liu et al., 2022)	99.11%	98.76%	98.82%	98.69%	86.24%	81.60%	80.26%	79.83%	28589128
RegNetX_400MF (Szegedy et al., 2015)	99.03%	98.78%	98.90%	98.79%	86.70%	82.00%	79.60%	79.32%	5157512
Mobilenet_v3_large (Howard et al., 2019)	98.12%	97.55%	97.22%	96.97%	85.05%	80.31%	78.74%	78.59%	5483032
EfficientNetB0 (Tan and Le, 2019)	98.10%	96.99%	97.11%	97.32%	84.27%	79.05%	77.32%	77.82%	8423848
PVT_Tiny (Wang et al., 2021)	99.04%	98.77%	98.63%	98.52%	86.04%	80.94%	79.45%	79.12%	12900072
EdgeNeXt_Small (Maaz et al., 2022)	98.98%	98.73%	98.67%	98.64%	85.95%	77.15%	76.03%	76.09%	7463368
**LMNet**	**99.23%**	**98.97%**	**98.94%**	**98.91%**	**87.27%**	**83.72%**	**82.91%**	**83.06%**	5679944

Bold font indicates the optimal model data.

Other results in **Table 9** indicate that models like ConvNeXt_tiny, RegNetX_400MF, and PVT_Tiny showed significant performance improvements after loading pre-trained weights. For instance, ConvNeXt_tiny’s test accuracy on the Plant Village dataset increased from 98.11% (as shown in **Table 8**) to 99.11%, and on the AI Challenger 2018 dataset, it saw a substantial increase from 81.96% to 86.24%. RegNetX_400MF’s test accuracy on the Plant Village dataset also rose from 97.06% to 99.03%. Similarly, PVT_Tiny’s test accuracy on the Plant Village dataset improved from 96.89% to 99.04%. This highlights the critical role of pre-training in enhancing the performance of these general-purpose models on target tasks.

However, not all models exhibited improvement after loading pre-trained weights. For example, Mobilenet_v3_large and EfficientNetB0 even showed a slight performance decrease on the AI Challenger 2018 dataset, which can be attributed to the domain discrepancy between the pre-training task and the downstream task. LMNet’s ability to achieve such excellent results without pre-training, especially surpassing SOTA models that rely on ImageNet pre-training, strongly suggests its architectural advantages in learning specific agricultural features. LMNet’s design is more focused on extracting unique, fine-grained visual patterns specific to plant diseases and agricultural scenes, such as subtle variations in leaf texture, the shape and color characteristics of lesions, and nuanced differences in crop growth stages.

Unlike general SOTA models (e.g., ConvNeXt_tiny), which are typically pre-trained on large-scale, diverse general datasets like ImageNet, the features they learn may be more geared towards general object recognition. When these general features are transferred to highly specialized agricultural domains, a “domain mismatch” issue can arise, leading to reduced effectiveness of some general features for agricultural tasks, or even introducing noise. LMNet, through its specially designed modules and connection mechanisms, likely avoids the interference of general features, directly and efficiently learning and encoding these domain-specific, highly discriminative features from agricultural data. This “from-scratch,” targeted learning approach enables LMNet to better adapt to the characteristics of agricultural images, thereby demonstrating excellent generalization ability and performance even without the need for external large-scale pre-training data.

### 3.6 Visualization verification

**Figure 10:**
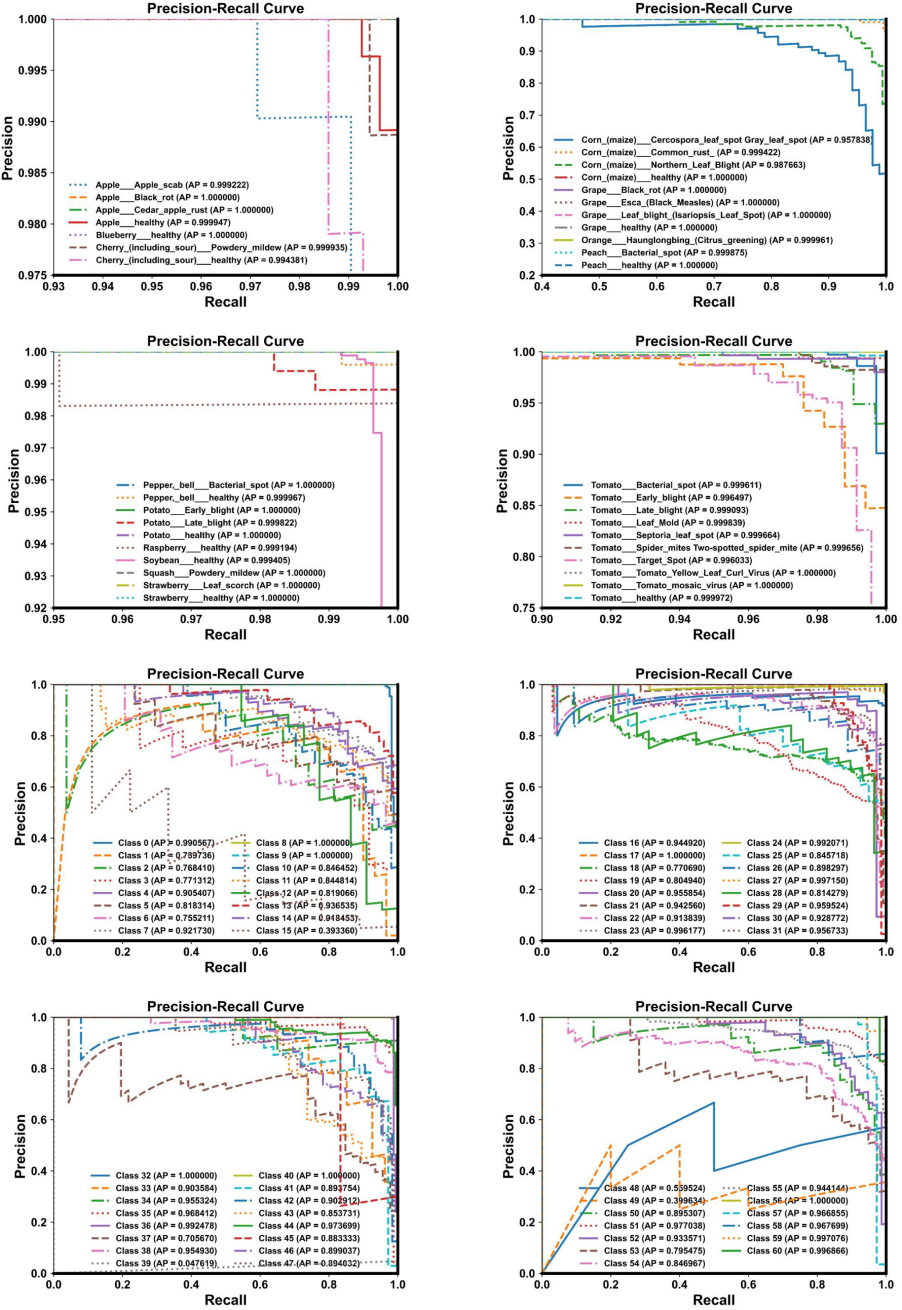
PR curve diagram. Up: Results on the plant village dataset. Down: Results on the AI challenger 2018 dataset.

**Figure 11:**
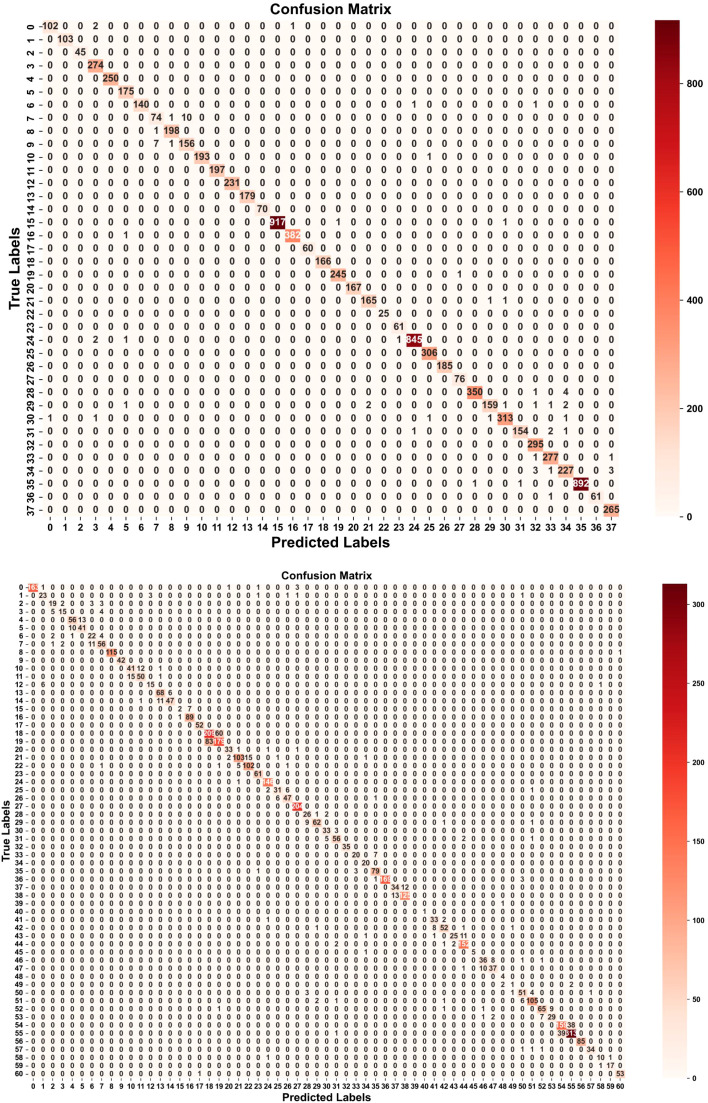
Confusion matrix diagram. Up: Results on the plant village dataset. Down: Results on the AI challenger 2018 dataset.

Although LMNet achieved the best performance on both the Plant Village dataset and the AI Challenger 2018 dataset, the performance gap between these two datasets reached as much as 11.96%. To investigate the reasons behind this discrepancy, we utilized Matplotlib to plot the ROC curves, PR curves, and confusion matrices for LMNet on these two datasets, visualizing its performance and classification capabilities across different categories. The results are presented in **Figures 9**–**11**. From **Figure 9**, it is evident that the ROC curves for LMNet on both datasets are very close to the top left corner, indicating a high true positive rate and a low false positive rate. Furthermore, the AUC values approach or equal 1, demonstrating LMNet’s robust ability to identify positive samples. In **Figure 10**, the PR curve for LMNet on the Plant Village dataset also approaches the top right corner, showing high precision and recall. However, the multiple PR curves for LMNet on the AI Challenger 2018 dataset do not converge towards the top right corner. Notably, labels 15, 39, 48, and 49 exhibit substantial deviations, suggesting that certain categories in the AI Challenger 2018 dataset have very few positive samples. This scarcity likely contributes to the poor performance of most classification models on this dataset. The confusion matrix depicted in **Figure 11** clearly illustrates the number of correctly classified samples for all categories by LMNet on both datasets. LMNet successfully identified the majority of disease images in the Plant Village dataset; however, it made classification errors for several categories in the AI Challenger 2018 dataset, including labels 18, 19, 54, and 55. This indicates that specific categories require attention in future model optimization efforts.

**Figure 12:**
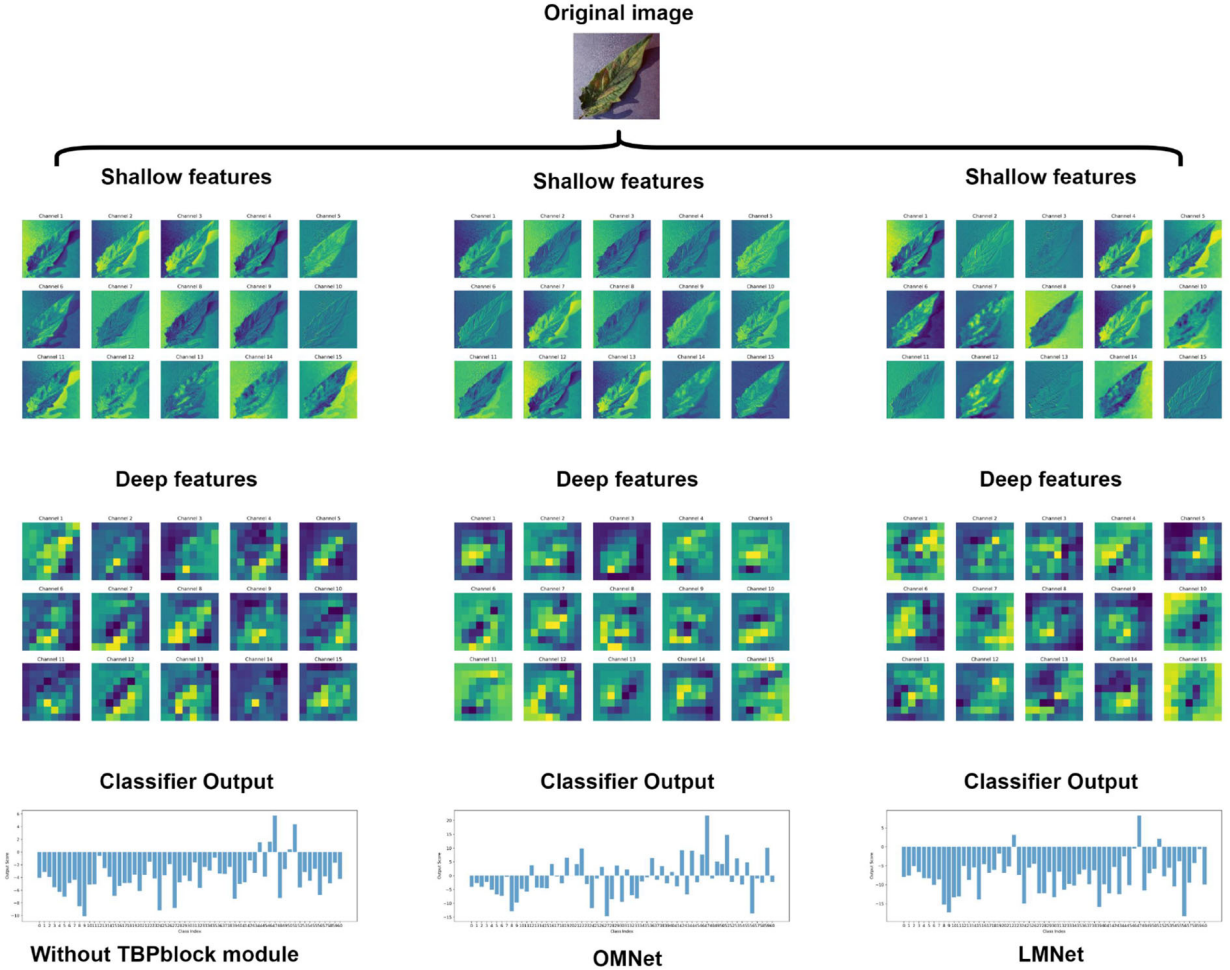
Comparison of feature extraction process visualizations.

**Figure 13:**
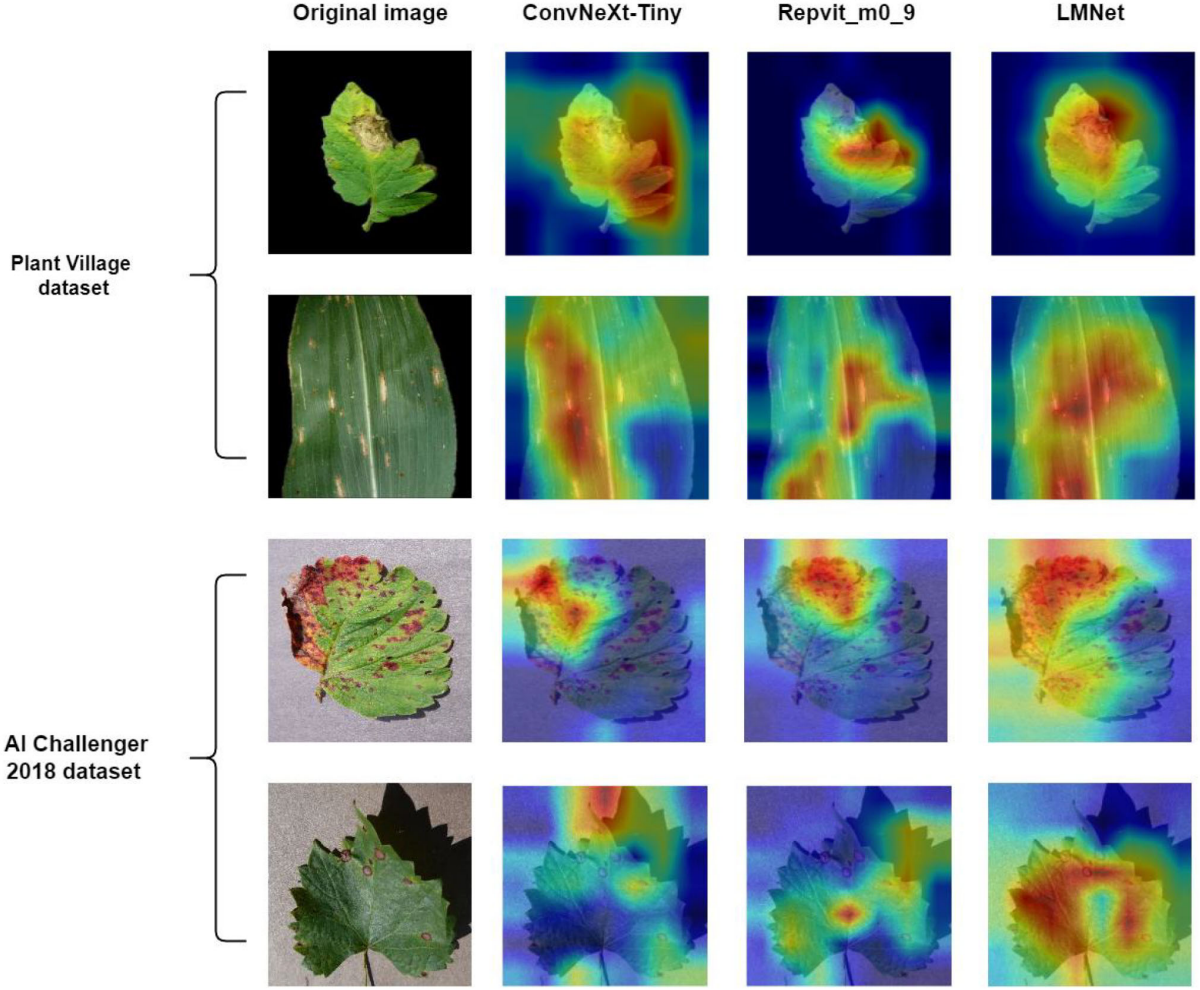
Comparison of Grad-CAM class activation mapping visualizations.

Finally, we used Grad-CAM (Selvaraju et al., [[NoYear]]) to visualize the feature extraction process of LMNet and the class activation maps of some exemplary images, as shown in **Figures 12**, **13**. In **Figure 12**, using the first 15 channels as an example, we observe that different models have similar extraction effects on the shallow features (such as shape and color) of diseased images during the feature extraction process. However, in the extraction of deeper features for plant leaf diseases, the model without the TBP block module fails to extract many abstract features due to its weaker multi-scale extraction capability. Both LMNet and OMNet can extract more in-depth abstract features. However, while OMNet extracts a large number of abstract features, some of its channel feature maps show overly smooth edges, such as channels 4, 5, 9, and 10. This could be due to a slight overfitting caused by OMNet’s higher complexity. When outputting the disease category, OMNet has the highest score for the correct category but fails to sufficiently suppress incorrect categories. In contrast, LMNet shows the most concentrated correct category scores and the most suppressed incorrect category scores, indicating its superior feature extraction capabilities. In **Figure 13**, we can see that LMNet identifies a larger area and with more precise localization of leaf diseases compared to ConvNeXt-Tiny and Repvit_m0_9. This suggests that LMNet performs better in identifying plant leaf diseases.

## 4 Discussion

We developed a new lightweight method for the task of plant leaf disease recognition, named the Lightweight Multi-Path Pruning Method (LMP-PM). LMP-PM flexibly performs selective lightweighting of the original model (OMNet) by adjusting the pruning parameter (N) and the path expansion factor (E) to meet the needs of different tasks. The OMNet has a complex structure that includes various modules, with the TBP block designed for multi-scale feature extraction to enhance recognition accuracy. Using LMP-PM, we lightweighted the original model to generate multiple variants and selected the model with a pruning parameter N of 4 and a path expansion factor E of 2, naming it LMNet. Subsequently, we conducted several ablation experiments on two large open-source plant leaf datasets: the Plant Village dataset and the AI Challenger 2018 dataset. The experimental results showed that, with fewer parameters, LMNet outperformed the original model and exceeded other similarly parametered lightweight models in multiple classification performance metrics. This further validates the effectiveness of our method.

The effectiveness of LMP-PM stems from its two-pronged approach, combining a pruning technique with a path expansion mechanism. This dual strategy enables the model to strike a precise balance between model size and accuracy. The pruning mechanism systematically reduces model complexity by decreasing the channel count of the input feature maps. As illustrated in **Table 2**, increasing the parameter N leads to a significant reduction in both parameters and FLOPs. However, excessive pruning alone can lead to a significant degradation in performance. For instance, a noticeable drop in accuracy is observed when N = 16 and E = 1. This suggests that while pruning effectively removes redundant information, it can also inadvertently discard critical features.

The path expansion mechanism counteracts this potential performance degradation by reintroducing model capacity and enhancing multi-scale feature learning. By transforming a single-path model into a multi-path architecture, it enables the model to learn more diverse and robust representations without significantly increasing the channel count for each individual path. As demonstrated in **Tables 3**-**5**, path expansion effectively mitigates the performance loss incurred by pruning and can even elevate model accuracy to surpass that of the original OMNet. For instance, the model configured with N = 4 and E = 2 achieves higher accuracy than OMNet, despite a significant reduction in both parameters and FLOPs. This suggests that the parallel paths empower the model to capture a richer set of features, thereby compensating for the information loss introduced by pruning.

Despite LMP-PM demonstrating significant advantages in lightweighting models for plant disease recognition, several notable limitations warrant future research. For instance, the optimal selection of pruning parameter (N) and path expansion factor (E) currently relies heavily on empirical experimentation. There is no automatic mechanism to determine the best N and E based on varying datasets or tasks. Consequently, identifying the optimal N and E for new tasks or datasets still necessitates a time-consuming search process. Furthermore, this research primarily focuses on global channel reduction. The lack of exploration into finer-grained pruning strategies, as well as an investigation into the impact of specific layers or blocks within the pruned network on model lightweighting and application effectiveness, represents an area for potential further optimization. Moreover, as depicted in **Figure 6**, increasing the path expansion factor (E) can lead to extended inference times. In latency-sensitive applications, this could potentially offset some of the lightweighting benefits. This suggests that while path expansion enhances accuracy, it may introduce computational overhead that requires further optimization for seamless real-time edge deployment. Finally, this study lacks experimental validation on actual mobile or embedded hardware. The method’s robustness to environmental noise, mixed symptoms, and domain shift was also not assessed. Addressing these issues in future work will significantly enhance the proposed method’s practical relevance and reliability.

We must explicitly state that OMNet was designed as a high-complexity, high-performance baseline model, integrating various structures such as residual modules, SE blocks, and our proposed TBP block. This design makes OMNet substantially large in terms of parameters and FLOPs. This is not accidental, but rather intended to serve as a rigorous stress test for the LMP-PM method. By applying lightweight processing to such a complex baseline, we aim to demonstrate that LMP-PM can not only simply compress models, but also effectively and significantly simplify a resource-intensive architecture while maintaining or even enhancing performance.

Transfer learning, particularly using ImageNet pre-trained weights, is a common and often effective method for accelerating model convergence and improving generalization capabilities on general tasks. However, for highly specialized domains like plant disease recognition, where subtle, fine-grained visual cues are critical, features learned from general datasets may not always be optimal. This can lead to domain shift issues, where general features may not transfer perfectly, or might even obscure domain-specific discriminative patterns.

It is in this context that LMNet achieved a test accuracy of 99.23% on the Plant Village dataset (outperforming the pre-trained ConvNeXt_tiny’s 99.11%, despite its larger parameter count), and ranked first among all compared models on the AI Challenger 2018 dataset with an accuracy of 87.27%. Crucially, all of this was achieved without any pre-training. This demonstrates that a carefully designed, lightweight architecture, optimized for the target domain from scratch, can effectively learn domain-specific features. These features exhibit comparable or even stronger discriminative power compared to those obtained through fine-tuning after general large-scale pre-training.

Therefore, LMP-PM’s unique value proposition extends beyond mere model compression. It offers a paradigm of architectural innovation and optimization, enabling the creation of highly efficient, domain-specific models. By systematically refining complex baseline models like OMNet, LMP-PM allows developed models to inherently adapt to the nuances of agricultural images, rather than relying on potentially mismatched general features. This provides an alternative pathway to achieving high performance in resource-constrained, domain-specific applications, where building optimized architectures from scratch may prove more effective than simply adapting pre-trained general models.

In the future, we aim to effectively apply LMP-PM in practical agricultural production to alleviate the application limitations posed by the large size of deep learning models. Additionally, we plan to further optimize LMP-PM based on its performance in real-world applications. This includes enhancing the adaptability of the lightweighted models for small hardware devices and researching the impact of pruning locations on the degree of model lightweighting and application efficacy. Building on this foundation, we will consider using LMP-PM for the lightweighting of more complex deep learning models, such as large-scale natural language processing models and vision models. Simultaneously, we will explore the effective integration of LMP-PM with deep learning optimization techniques. Knowledge distillation serves as an efficient lightweighting strategy by transferring features extracted from a high-performing, complex teacher model to a lower-complexity student model, thereby improving the performance of the student model (Cai et al., 2024; Wang J. et al., 2024). LMP-PM can provide multiple student models for knowledge distillation, further advancing the development of model lightweighting. Additionally, multimodal fusion techniques can combine data from different modes or sources (such as text, images, and audio) to acquire more comprehensive information, thereby enhancing the performance and effectiveness of tasks (Liu et al., 2023; Chen et al., 2024; Li J. et al., 2024). LMP-PM can facilitate the lightweighting of multimodal backbone networks while providing multiple path models for various input modalities, achieving an organic integration with multimodal tasks.

## Data Availability

The original contributions presented in the study are included in the article/supplementary material. Further inquiries can be directed to the corresponding author.
